# Generalize Robot Learning From Demonstration to Variant Scenarios With Evolutionary Policy Gradient

**DOI:** 10.3389/fnbot.2020.00021

**Published:** 2020-04-21

**Authors:** Junjie Cao, Weiwei Liu, Yong Liu, Jian Yang

**Affiliations:** ^1^Institute of Cyber Systems and Control, Zhejiang University, Hangzhou, China; ^2^China Research and Development Academy of Machinery Equipment, Beijing, China

**Keywords:** learning from demonstration, generalization, exploration, reinforcement learning, evolutionary algorithms

## Abstract

There has been substantial growth in research on the robot automation, which aims to make robots capable of directly interacting with the world or human. Robot learning for automation from human demonstration is central to such situation. However, the dependence of demonstration restricts robot to a fixed scenario, without the ability to explore in variant situations to accomplish the same task as in demonstration. Deep reinforcement learning methods may be a good method to make robot learning beyond human demonstration and fulfilling the task in unknown situations. The exploration is the core of such generalization to different environments. While the exploration in reinforcement learning may be ineffective and suffer from the problem of low sample efficiency. In this paper, we present Evolutionary Policy Gradient (EPG) to make robot learn from demonstration and perform goal oriented exploration efficiently. Through goal oriented exploration, our method can generalize robot learned skill to environments with different parameters. Our Evolutionary Policy Gradient combines parameter perturbation with policy gradient method in the framework of Evolutionary Algorithms (EAs) and can fuse the benefits of both, achieving effective and efficient exploration. With demonstration guiding the evolutionary process, robot can accelerate the goal oriented exploration to generalize its capability to variant scenarios. The experiments, carried out in robot control tasks in OpenAI Gym with dense and sparse rewards, show that our EPG is able to provide competitive performance over the original policy gradient methods and EAs. In the manipulator task, our robot can learn to open the door with vision in environments which are different from where the demonstrations are provided.

## 1. Introduction

Hand-engineering of a controller is the basic approach to make robots autonomous for a certain task. For tasks whose execution depends on the circumstances of the environment or the interaction with human, robots must handle complex perception. However, the hand-engineering method for such task is tired, especially for vision-based tasks which are exceptionally difficult. In order to overcome those problems, a significant research area in contemporary robotics centers on approaches where the robot controller is learned rather than programmed (Rahmatizadeh et al., 2017). Learning from demonstration (LfD) allows humans to demonstrate a specific task to the robot without having any knowledge about the robot's dynamic model or programming the control commands. One direct approach to learning from demonstration is Behavior Cloning, where human demonstrates the desired behavior to the robot – as supervisory signals of what the robot should do in the same states. However, the demonstrations are expensive to acquire and it is difficult to acquire complex manipulation skills just from demonstration.

On the other hand, the learned behavior is restricted to specific environment where human provide demonstrations. In order to generalize robot learning from demonstration to other scenarios, robot should to explore in the new situations. Reinforcement learning, learning to control through exploration by trial and error, provides a promising method for robot learning with generalization. For exploration, novel control policies are needed to gain diverse experience that is informative about the environment. The diverse experience is used to optimize the policies in return. In exploitation, the learning procedure exploits the good policy to collect state-action pairs with high rewards and further improve the performance of the good policy. In contrary to the exploration that may find best policy slowly and globally, exploitation aims to optimize the policy more efficiently and locally. To generalize robot learning and be sample efficient, preventing the mechanical wear, it usually needs to trade-off between exploration and exploitation in interaction with the real world. Proposing efficient and effective robot learning method has always been the hot spot in robotics.

Random perturbation of the agent's action is the classic method to induce novel behaviors, such as ϵ-greedy for Q-learning (Sutton and Barto, 1998) and policy gradient with action noise (Silver et al., 2014). However, action noise is usually independent of states and in fact is local perturbation, which generates unsmooth trajectories and is unlikely to produce various large-scale behavioral patterns for effective exploration (Osband et al., 2017). Recent works (Fortunato et al., 2017; Plappert et al., 2017; Gangwani and Peng, 2018) have show that exploration with parameter noise outperforms action noise, especially in tasks where the reward is sparse. Instead of promoting exploration with parameter perturbation, a lot of other explore strategies rely on the state novelty to increase the diversity of experience and obtain outstanding results in computer games (Bellemare et al., 2016; Houthooft et al., 2016; Pathak et al., 2017; Tang et al., 2017).

Evolutionary Algorithms (EAs) have been successfully used to optimize policy represented with neural network (Such et al., 2017) by perturbing and searching directly in the policy parameter space. The policy parameter evaluation of EAs is based on the cumulative reward received in the whole episode. Thus, EAs optimize the policy with more comprehensive insight and can solve the sequential decision making problems that have sparse reward signals.

Policy gradient methods are usually used to search best policy greedily, promoting better exploitation. As the original version of policy gradient algorithm, REINFORCE (Williams, 1992) tends to be of high variance due to the gradient estimation with Monte Carlo method. Actor-critic methods (Mnih et al., 2016) use the value function to reduce the variance and improve the performance of policy gradient. In order to improve the accuracy of gradient estimation and stabilize the learning procedure, Schulman et al. (2015, 2017) constrained the step size of policy gradient descent within a local area of previous policy. Results from previous research show that policy gradient methods are sample-efficient by taking advantage of the temporal structure of the experience. However, those policy gradient methods with action noise tend to converge to the local optimum, especially when the exploration is insufficient.

In this paper, we introduce Evolutionary Policy Gradient (EPG), incorporating the policy gradient methods with Evolutionary Algorithms (EAs). In our framework of evolutionary algorithm, population-based approach generates different policies with parameter perturbation to improve exploration. With demonstration guiding the evolutionary procedure, our EPG guides the robot to behave similarly to the demonstrations, which prevent robot exploring in unpromising area. By selecting elites based on the fitness metric that evaluates the cumulative reward of an entire episode, EPG pushes the next generation of policy toward regions that lead to higher probability of task accomplishment in the current situation. Thus, our EPG can explore to generalize with diverse policies and also prevent the ineffective exploration with demonstration guiding.

## 2. Related Work

In recent years, Learning from demonstration (LfD) has been successfully used in the field of robotics for applications in autonomous helicopter maneuvers (Abbeel et al., 2010), playing table tennis (Calinon et al., 2010), multi-task manipulation (Rahmatizadeh et al., 2017), and deformable object manipulation (Matas et al., 2018). A major challenge in LfD is to extend these demonstrations to unseen situations (Argall et al., 2009). To mitigate this problem, one obvious way is to acquire a large number of demonstrations covering as many situations as possible. With limited demonstration data, Sylvain et al. (2007) and Calinon et al. (2009) propose to hand-engineer task-specific features. Different from those methods we use smaller number of demonstrations, but change the learning model by exploring in new situations to generalize better. We resort to reinforcement learning and evolutionary algorithm to learn from demonstration and generalize to unseen situations. Rajeswaran et al. (2018) also combined reinforcement learning with demonstration to accomplish complex dexterous manipulation tasks in the same environment as where the demonstrations come from. While our goal is to generalize the learning behavior with the demonstration from one specific situation to behave well in other situations. Nair et al. (2017) avoided invalid exploration in reinforcement learning with demonstration. Our experiments also present such benefits of demonstration in comparison with pure reinforcement learning, even though the demonstrations in our experiments is coming from environment with different configurations. We have not find any previous works focus on generalizing the skill learning from demonstration to other situations. But we think the reinforcement learning after imitation learning can accomplish such goal and we also compare our algorithm with such method.

Exploration during learning from demonstration is the core of our work. And the lack of effective exploration are also the major challenges in Reinforcement Learning, especially in the environments with long time horizon and sparse reward. Many explore strategies, which rely on the state novelty, have been proposed to improve the diversity of trajectories (Bellemare et al., 2016; Houthooft et al., 2016; Pathak et al., 2017; Tang et al., 2017). One common point of those works is the need of complex supplementary structures to estimate the novelty which will introduce some additional sensitive hyper parameters and are suited mainly for exploration in video games. However, our EPG, which explore with the framework of EAs, is more general with an easy modification to the original policy gradient method.

ES and PGPE which explore with parameter perturbation can be regarded as Evolutionary Algorithm and are scalable to be implemented in parallel. Salimans et al. (2017) have demonstrated that ES is suited for the problem with long time horizon and delayed reward and does not need the approximation for the value function. PGPE (Sehnke et al., 2010) performs gradient based search in parameter space with low variance and is similar to ES. Wang et al. (2017) improve PGPE with EM-based policy exploration and an adaptive mechanism. To further improve exploration in ES, especially on sparse or deceptive Reinforcement Learning problems, Conti et al. (2017) hybrid novelty search and quality diversity algorithms with ES. Unlike ES, NES, and PGPE which are gradient based methods, recently, Such et al. (2017) evolve the weights of a deep neural network with Genetic Algorithm to solve RL problems. By comparing DDPG with CMA-ES, de Broissia and Sigaud (2016) conclude that policy gradient methods are significantly more sample-efficient than ES. And it is the same for other EAs, without taking advantage of temporal structure in the trajectories. Our EPG incorporates the policy gradient into the framework of EAs to exploit the sample efficiency of policy gradient methods.

Recent works (Fortunato et al., 2017; Plappert et al., 2017) proposed to explore by adding noise to the parameter space and optimizing policy with gradient descent. Their results have shown that parameter perturbation can successfully be combined with reinforcement learning algorithms and often lead to improved performance compared to adding noise in action space. Similar to those works, our EPG also combines parameter perturbation with policy gradient methods. To further improve the exploration and parallelizability, our EPG resorts to the framework of EAs. Inspired by the Genetic Algorithms, Gangwani and Peng (2018) mutate the policy with policy gradient methods. However, without perturbing policy parameter vector during policy evolution, the diversity of policies and exploration in their method are limited. Our EPG retains the major benefits of the recent works, and the whole procedure can be approximated to the optimization of an objective function that evaluates the Gaussian distribution of policy parameter.

## 3. Background

### 3.1. Learning From Demonstration (LfD) With Behavior Cloning

In recent years, LfD was successfully used in the field of robotics. Behavior cloning, as the simplest learning from demonstration method, can be performed using standard, efficient supervised learning methods. Compared with reinforcement learning methods that learn from scratch, behavior cloning requires fewer interactions with the environment.

Provided with the observation-action pairs, behavior cloning can fit a stochastic policy with supervise learning, mapping observations to distributions of action directly, just by maximizing the log likelihood of the demonstrated actions:

(1) $$\begin{array}{r} L=-E_{\left(s,a\right)\sim demo}\left[\log\pi \left(a|s\right)\right]. \end{array}$$

where (*s, a*) represents the state-action pair from demonstrations, *E* represents expectation over (*s, a*) and π is the stochastic policy to be optimized.

### 3.2. Policy Gradient and Explore With Action Noise

Reinforcement Learning (RL) is popular in solving sequential decision making problems where a robot interacts with an environment, sequentially choosing an action *a*_*t*_ according to a policy π(*a*|*s*) based on the state *s*_*t*_ at time *t*. After taking the action *a*_*t*_, state *s*_*t*_ transforms to the next state *s*_*t*+1_. And the robot receives a scalar reward *r*(*s*_*t*_, *a*_*t*_) from the environment. In the Markovian environment, the probability distribution over the next state *s*_*t*+1_, called transition probability, is satisfying Markov property, i.e., *s*_*t*+1_~*p*(*s*_*t*+1_|*s*_*t*_, *a*_*t*_). The objective of robot learning with RL is to obtain a policy π which maximizes the expected discounted cumulative reward, i.e., $J\left(\pi \right)=E_{\tau }\left[\underset{t}{\sum }\gamma^{t}r\left(s_{t},a_{t}\right)\right]$, where γ is the discounted factor that trade-off between shorter and longer term rewards. Solving such problem can be modeled as Markov Decision Process (MDP).

Policy Gradient method is one kind of reinforcement learning algorithms. For exploration in action space, stochastic policy samples from a Gaussian distribution $\pi_{\theta }\sim N\left(\mu \left(s\right),\sigma {\left(s\right)}^{2}I\right)$ with μ(*s*) and σ(*s*) parameterized by θ, at each time step. Stochastic policy gradient methods maximize the expected cumulative reward by estimating the performance gradient ∇_θ_*J*(π_θ_) based on the Stochastic Policy Gradient Theorem (Sutton et al., 2000). For deterministic policy gradient methods, such as DPG (Silver et al., 2014) and DDPG (Lillicrap et al., 2015), the critic estimates the state-action value function *Q*(*s, a*) using off-policy data which is sampled with a noisy policy. The noisy policy improves the exploration by adding additive action noise to deterministic policy: ${\hat{\pi }}_{\theta }\left(s\right)=\pi_{\theta }\left(s\right)+w$, where *w* represents the action noise with its variance annealing to trade-off between exploration and exploitation.

### 3.3. Policy Search With Evolutionary Algorithms

Most real-world problems can be modeled as MDP in which agents or robots only receive a reward signal after a series of actions. In the MDP where rewards are sparse, it is difficult to associate actions with rewards. This situation is often denoted as the temporal credit assignment problem (Sutton and Barto, 1998).

Inspired by natural selection, Evolutionary Algorithms (EAs) are a series of black box optimization methods which are heuristic search procedures with several operators: new solution generation, mutation, selection, crossover and so on. Evolutionary Algorithms for sequential decision making problems are invariant to sparse rewards with long time horizons (Fortunato et al., 2017). Population-based approach in EAs has the advantage of promoting exploration, by parameter perturbation (mutation). The redundancy in a population and the selection of elites improve the robustness and stability of the heuristic search procedure. In computation complexity, EAs outperform back propagation methods in optimizing neural network with only forward evaluation of the parameter. Because of these merits of EAs, a number of recent research in RL problems have used EAs as an alternative to standard RL algorithms. Such et al. (2017) use genetic algorithms (GAs) to train deep neural networks for policy search. Conti et al. (2017) and Salimans et al. (2017) indicate that evolutionary strategies (ES) is scalable alternative to Reinforcement Learning and can improve exploration in RL.

In policy gradient methods, after sampling on each time step, the gradient is calculated by differentiating the policy with respect to the parameters. However, the derivative of the policy may not exist or be difficult to calculate. And sampling from the noisy policy on each time step leads to the noisy gradient estimation. Some EAs address such variance problem by replacing the random action sampling with parameter vector sampling, like natural evolution strategies (NES) (Wierstra et al., 2008) and policy gradients with parameter-based exploration (PGPE) (Sehnke et al., 2010). These algorithms represent the population with a probability distribution *p*(θ|ρ) over policy parameters θ, where ρ is the parameter of the distribution. Instead of sampling action at each time step with stochastic policy, PGPE samples a policy parameter vector from *p*(θ|ρ) to construct a deterministic policy, from which actions are taken. So PGPE addresses the variance in trajectory and noisy gradient problems by generating an entire trajectory with only one parameter vector sampled before exploration. The objective function to be maximized by searching ρ with stochastic gradient ascent is the expected cumulative reward over all parameter vectors:

(2) $$\begin{array}{r} J\left(\rho \right)=\int_{\theta }\int_{\tau }p\left(\tau ,\theta |\rho \right)R\left(\tau \right)d\tau d\theta , \end{array}$$

where *R*(τ) represents the cumulative reward in a trajectory τ. Differentiating this objective function with respect to probability distribution parameter ρ, the gradient can be estimated by sampling θ from *p*(θ|ρ), then running the policy with parameter θ to generate trajectory τ, which submits τ~*p*(τ|θ). PGPE will choose Gaussian distribution as the policy parameter probability distribution, i.e., $p\left(\theta |\rho \right)=N\left(\mu ,\sigma^{2}I\right)$ with ρ = [μ, σ]. Optimizing μ and keeping σ as a constant, PGPE (Sehnke et al., 2010) reduces to evolution strategies (ES) (Salimans et al., 2017).

The ES, NES, and PGPE, introduced above, perform stochastic gradient descent with the calculation of gradient similar to the finite-difference methods, and are gradient-based algorithms. As another kind of classical Evolutionary Algorithms (EAs), a truly gradient-free method, Genetic Algorithm can also train deep neural networks for policy to solve the challenging sequential decision making problem. However, EAs for policy search do not exploit the information of each state-action pair in trajectories which make the policy gradient algorithms more sample-efficient. Thus, EAs need more samples of environment interaction (de Broissia and Sigaud, 2016).

## 4. Method: Evolutionary Policy Gradient

Based on the framework of EAs, in this work, we define the policy with parameter vector θ and fixed neural network structure as individual. The mutation operator in our EPG includes random mutation and optimal mutation. EPG selects some elites, i.e., good policy parameter vectors according to the evaluation of the fitness function. As most EAs for reinforcement learning problems, the fitness function in EPG is defined as the average cumulative rewards of several episodes, where the rewards are received by robot after accomplishing tasks. Usually, EAs perform crossover directly in parameter space (Floreano et al., 2008) to increase the diversity of population. Instead, the crossover operator in our EPG combines the elites in the action distribution of the policies. And the combination is highly relied on the demonstration guided imitation learning.

Evolutionary algorithms (EAs), usually regarded as black box optimization processes, are heuristic and lack the theoretical guarantee. Although EPG is based on the framework of EAs, in this section, we will first derive the stochastic policy gradient method for the perturbed policies in mathematics and then propose our improvement to fit the framework of EAs with demonstrations guiding its procedure.

### 4.1. Optimization of Perturbed Policies With Policy Gradient

To achieve structured exploration, EPG will perturb current policy parameter vector to form a set of policies by applying additive Gaussian noise to the parameter vector of the current policy: $\theta =\theta^{\prime }+N\left(0,\sigma^{2}I\right)$. The policy parameter perturbation is actually sampling parameters from the probability distribution *p*(θ|ρ) with ρ = [μ, σ], and μ is equal to the current policy parameter vector θ′. EPG optimizes the mean of the probability distribution of the parameters with gradient descent, while initiates the variance of the distribution and anneals it during the training procedure. The initial σ represents the capability of exploration and can be tuned according to the sparsity of rewards. The objective function here is the same as that of PGPE and ES (i.e., Equation 2).

Now, we derivate the gradient of the same objective *J*(ρ) in a different way as in PGPE (Sehnke et al., 2010). After rewriting equation (2) in the discrete format, noting τ is conditionally independent of ρ given θ, so *p*(τ, θ|ρ) = *p*(τ|θ)*p*(θ|ρ), we have:

(3) $$\begin{array}{r} \begin{array}{c} J\left(\rho \right)=\underset{\epsilon \sim N\left(0,I\right)}{\sum }\underset{\tau }{\sum }p\left(\tau |\epsilon \sigma +\mu \right)R\left(\tau \right). \end{array} \end{array}$$

By sampling ϵ~N(0,I) to construct policy parameters and executing the policy to generate τ, the gradient of *J*(ρ) with respect to probability distribution parameter ρ can be calculated:

(4) $$\begin{array}{r} \nabla_{\rho }J\left(\rho \right)=\frac{1}{N}\underset{\begin{array}{c} \epsilon \sim N\left(0,I\right)\\ \tau \sim p\left(\tau |\epsilon \sigma +\mu \right) \end{array}}{\sum }\nabla_{\rho }\log p\left(\tau |\epsilon \sigma +\mu \right)R\left(\tau \right). \end{array}$$

For finite horizontal Markov decision process (MDP) with trajectory τ = [*s*_1:*T*_, *a*_1:*T*_], in which *s*_1:*T*_, *a*_1:*T*_ represents the sequence of state and action pairs, we have:

(5) $$\begin{array}{r} \log p\left(\tau |\theta \right)=\overset{T}{\underset{t=0}{\sum }}\log p\left(s_{t+1}|s_{t};a_{t}\right)+\log\pi_{\theta }\left(a_{t}|s_{t}\right). \end{array}$$

Substituting equation (5) into (4) and noting that we only optimize μ, we replace ρ with μ and denote π_ϵσ+μ_ with π_μ_ for clarity:

(6) $$\begin{array}{r} \nabla_{\mu }J\left(\mu \right)=\frac{1}{N}\underset{\epsilon \sim N\left(0,I\right)}{\sum }\overset{T}{\underset{t=0}{\sum }}\nabla_{\mu }\log\pi_{\mu }\left(a_{t}|s_{t}\right)R\left(\tau \right). \end{array}$$

To reduce the variance of the gradient estimation, the cumulative reward *R*(τ) can be substituted with the advantage function $A^{\pi_{\mu }}\left(s_{t},a_{t}\right)$, which represents the improvement in cumulative reward obtained so far by taking action *a*_*t*_ in state *s*_*t*_. According to the previous work (Mnih et al., 2016), we calculate the advantage function with an approximated value function and the obtained cumulative reward.

Proximal policy optimization algorithms (PPO) (Schulman et al., 2017) optimizes a “surrogate” objective function including a penalty term to constrain the size of the policy update. The updating equation for optimizing equation (2) with PPO can be derived as:

(7) $$\begin{array}{rcl} \nabla_{\mu }J\left(\mu \right) & = & \frac{1}{N}\underset{\epsilon }{\sum }[\underset{t}{\sum }A^{\pi_{\mu_{old}}}\left(s_{t},a_{t}\right)\frac{\nabla_{\mu }\pi_{\mu }\left(a_{t}|s_{t}\right)}{\pi_{\mu_{old}}\left(a_{t}|s_{t}\right)}\\ - & \beta KL\left[\pi_{\mu_{old}}\left(\cdot |s_{t}\right),\pi_{\mu }\left(\cdot |s_{t}\right)\right], \end{array}$$

where π_μ_, π_μ_*old*__ represent the current policy and the policy after previous iteration, respectively. *KL*[π_μ_*old*__(·|*s*_*t*_), π_μ_(·|*s*_*t*_)] is the Kullback–Leibler divergence between the action distributions of the two policies, and β works as the coefficient of the penalty term.

### 4.2. The Framework of EPG

Our EPG algorithm is derived and approximated from the optimization of perturbed policies with policy gradient methods, by adding some heuristic of EAs. As EAs differ in how to perform those operators, we define the mutation, selection and crossover to form the framework of our EPG.

For exploration, EPG generates a population of *N* individuals (policy parameter vector θ_*i*_) by parameter perturbation, which applies additive Gaussian noise to the parameter vector of the current policy: θ_*i*_ = μ+σϵ, where ϵ~N(0,I). The parameter perturbation in EPG can be regarded as the random mutation of the individual. After executing each perturbed policy (individual) for several episodes, the fitness can be evaluated by averaging the cumulative rewards received during those episodes. Then EPG performs truncation selection, where the top *n* individuals become the elites. For each elite individual, EPG optimizes the policies with policy gradient algorithms, which can be regarded as optimal mutation. Those policy gradient optimization processes can be implemented in parallel to accelerate the training procedure. After the optimal mutation, it is easy to combine those elite optimal policies to one policy by averaging their parameter vectors.

Using ∇_μ_*J*(μ), Equation (7), to update the mean of policy parameter vector μ, noting θ_*i*_ = ϵ_*i*_σ+μ and $\epsilon_{i}\sim N\left(0,I\right)$, we get:

(8) $$\begin{array}{r} \begin{array}{c} \hat{\theta }=\mu +\frac{1}{N}\overset{N}{\underset{i=1}{\sum }}\underset{t}{\sum }A^{\pi_{\theta_{i}}}\left(s_{t},a_{t}\right)\nabla_{\mu }\log\pi_{\epsilon_{i}\sigma +\mu }\left(a_{t}|s_{t}\right)\\ \approx \frac{1}{n}\overset{n}{\underset{i=1}{\sum }}\left[\mu +\epsilon_{i}\sigma +\underset{t}{\sum }A^{\pi_{\theta_{i}}}\left(s_{t},a_{t}\right)\nabla_{\mu }\log\pi_{\epsilon_{i}\sigma +\mu }\left(a_{t}|s_{t}\right)\right]\\ =\frac{1}{n}\overset{n}{\underset{i=1}{\sum }}\left[\theta_{i}+\Delta \theta_{i}\right]. \end{array} \end{array}$$

where Δθ_*i*_ is calculated with policy gradient methods with regard to policy parameter vector θ_*i*_. Equation (8) shows that the framework mentioned above is actually some kind of approximation to the gradient descent procedure of optimizing objective function (2).

The selection of *n* elites out of the population by choosing *n* best policies is a technique called elitism in Evolutionary Algorithms (EAs). The mutation in EPG includes random mutation by policy parameter perturbation and optimal mutation by policy optimization with policy gradient. The crossover in the parameter space gives few reasonable explanation. We resort to the ensemble learning to crossover those elite policies by learning a classifier to choose which elite policy to take action at every time step. Combining the classifier and the optimized elite polices, we get a compound policy. In order to generate the child policy for the next generation, we first initialize the child policy with the average parameter vector of elite polices, then minimize the KL divergence between the action distributions of the child policy and the compound policy. The overall crossover operator in our EPG can be regarded as the crossover in the action space, which seems to be more reasonable since the output of policy is action. The crossover operator is also equivalent to imitation learning after ensemble, combining the diverse elite policies. The objective of imitation learning can be augmented with that of behavior clone to guide the evolutionary process with demonstration. After the crossover, we get one individual (child policy) on which random mutation (parameter perturbation) can be implemented. It means a new generation (iteration) begins.

**Figure 1:**
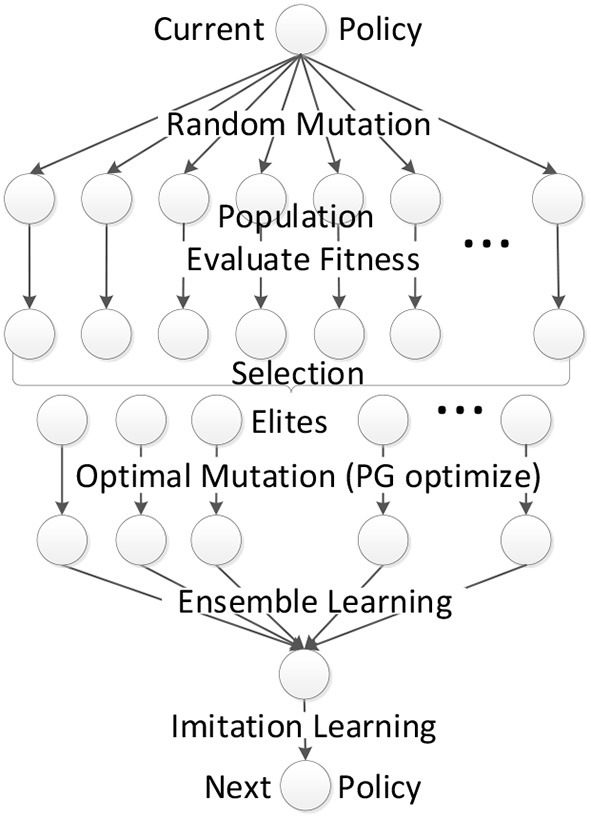
Framework of EPG.

The whole EPG algorithm is scalable to be implemented in parallel in different computer nodes with different random seeds. Algorithm 1 illustrate the procedure in one single node of our EPG. Figure 1 illustrates the complete learning procedure of EPG. In the rest of this section, the mutation and crossover operators will be detailed.

**Table:** **Algorithm 1:** The procedure in node *i* of EPG.

1: **repeat**
2: Random mutate (perturb) π_θ_ → π_θ_*i*__;
3: Execute policy π_θ_*i*__ for several episodes to collect rollout trajectories τ;
4: Optimize policy with policy gradient: $\pi_{\theta_{i}}\to {\hat{\pi }}_{\theta_{i}}$;
5: Send the parameters of ${\hat{\pi }}_{\theta_{i}}$, cumulative rewards *R*_*i*_ and visited states *S*_*i*_ in τ to every other nodes;
6: Calculate *fitness*(π_θ_*j*__) by averaging *R*_*j*_ in every nodes;
7: Select optimized elite policies ${\hat{\pi }}_{\theta_{j}}$ according to *fitness*(π_θ_*j*__), *j*∈[1, *n*];
8: Child policy $\pi_{\theta }^{\prime }\leftarrow Crossover\left({\hat{\pi }}_{\theta_{1:n}},S_{1:n},R_{1:n},demo\right)$ ;
9: **until** *k* times of evolution loop.

### 4.3. Mutation Operator in EPG

Similar to most EAs in solving sequential decision making problems, EPG operates mutation by perturbing current policy to generate a population of policies. Parameter perturbation, called random mutation, is implemented on stochastic policy instead of deterministic policy as policy gradients with parameter-based exploration (PGPE) (Sehnke et al., 2010). The stochastic policy outputs the mean and variance of a Gaussian distribution from which action is sampled. The output variance of stochastic policy and the variance of parameter perturbation determine the exploration in action and parameter space, respectively. By combining the exploration both in action space and parameter space, EPG can avoid local minima more easily.

After selecting elites from the population, EPG modifies each elite with policy gradient methods in parallel, which is regarded as optimal mutation. The policy gradient is estimated with the rollout samples collected by stochastic policy in environment with different random seeds, thus induces randomness for elite policies update. So the optimal mutation operator still maintains sufficient diversity of the population and good exploration in the state space. The optimal mutation improves EPG's efficiency in the usage of sampling data, by taking advantage of the powerful gradient descent method and reusing the rollout samples generated for elite selection.

### 4.4. Crossover Operator in EPG

The crossover operator mixes parent policies and produces a new child policy. Those policies have identical network architectures. Floreano et al. (2008) produced the child policy by averaging the parameters of the parent policies, which called average crossover. Such crossover operation in parameter space dose not has steady performance, sometimes producing a worse policy than the policies before averaging. In our EPG framework, the finite number of population *N* usually results in the underestimation of policy gradient (6). It is more intuitive to crossover the policy in the distribution of action, since the output of policy is a distribution of action dependent on the state.

In our framework of EPG, every elite policies are trained independently with different random seeds, thus have distinct state distributions encountered. So we propose to first combine elite policies with ensemble learning, by learning a classifier $\pi_{c}\left({\hat{\pi }}_{\theta_{j}}|s\right)$ to choose one elite policy ${\hat{\pi }}_{\theta_{j}}$ according to their visited state *s*. The intuition is that given a state we choose the elite policy, which encountered this state more often before, to take action. The objective of the classifier is weighted maximum likelihood:

(9) $$\begin{array}{r} J^{ML}=-\underset{s}{\sum }\left[\omega_{s}\log\underset{j}{\sum }\pi_{c}\left({\hat{\pi }}_{\theta_{j}}|s\right)1_{s\in \tau_{j}}\right], \end{array}$$

where *1*_*s*∈_τ__*j*__ is the indicator function and ω_*s*_ is the weight for state *s*, been the same in one trajectory. The formulation of ω_*s*_ is detailed in Algorithm 2, where *R*_*j*_ represents the set of cumulative rewards of several trajectories generated with elite *j* and *R*_*jk*_ is the *k*'th element of *R*_*j*_. With the classifier and the elite policies, we get a compound policy: first select an elite policy and then take action according to it.

**Table:** **Algorithm 2:** Crossover operator in node *i* of EPG.

**Require:** ${\hat{\pi }}_{\theta_{j}},S_{j},R_{j},j\in \left[1,n\right]$ of elite policies; Demonstrations from one fixed environment: (*s, a*)~*demo*
**Ensure:** Child policy $\pi_{\theta }^{\prime }$, generalized to a different situation as in demonstrations;
1: For *s* in the *k*'th trajectory of elite *j*: $\omega_{s}=\frac{R_{jk}-\min R_{j}}{\max R_{j}-\min R_{j}}$;
2: Send the gradient of (9): $\nabla_{c}J_{j}^{ML}$ to other nodes;
3: Train $\pi_{c}\left({\hat{\pi }}_{\theta_{j}}|s\right)$ with gradient: $\frac{1}{n}\underset{j}{\sum }\nabla_{c}J_{j}^{ML}$;
4: Combine classifier and elite policies to form π_*exp*_(·|*s*)
5: Average $\theta =\frac{1}{n}\overset{n}{\underset{j=1}{\sum }}\theta_{j}$ to initialize child policy $\pi_{\theta }^{\prime }$;
6: Initialize data set: $\left\{\hat{\tau_{i}}\right\}$;
7: **repeat**
8: Generate one trajectory τ with $\pi_{\theta }^{\prime }$;
9: Aggregate data set $\left\{\hat{\tau_{i}}\right\}$ with τ;
10: Calculate the gradient of (10): $\nabla_{\theta }J_{j}^{IL}$ with $\left\{\hat{\tau_{i}}\right\}$;
11: Send $\nabla_{\theta }J_{j}^{IL}$ to other nodes;
12: Update $\pi_{\theta }^{\prime }$ with gradient: $\frac{1}{n}\underset{j}{\sum }\nabla_{\theta }J_{j}^{IL}$;
13: **until** *t* times of imitate iterations.

To inherit the essence of the optimized elite policies, our EPG crossovers them by imitating the compound policy, also called expert, with imitation learning. The expert, represented as $\pi_{exp}=\pi_{c}\left({\hat{\pi }}_{\theta_{j}}\left(\cdot |s\right)|s\right)$, includes a classifier and several elite policies. Imitation learning is a method that develops new policy by mimicking expert's behaviors. The imitation is implemented in the action distribution by minimizing the KL divergence between the action distributions of the child policy and the compound policy: *KL*[π_θ_, π_*exp*_]. Then, the resulting π_θ_ is an approximate Gaussian I-projection of π_*exp*_ which works as a good guiding distribution. To improve the sample efficiency, we initialize the child policy with the same architecture as elite policies and the average elite policies' parameter vectors. Moreover, in order to direct the policy evolution to more likely accomplish the task, especially at the beginning of exploration, we augment the objective of KL divergence with the negative log likelihood of demonstrated actions (Equation 1), resulting the objective function to be minimized:

(10) $$\begin{array}{r} J^{IL}\left(\theta \right)=\underset{s}{\sum }KL\left[\pi_{\theta }\left(\cdot |s\right),\pi_{exp}\left(\cdot |s\right)\right]-\lambda E_{\left(s,a\right)\sim demo}\left[\log\pi_{\theta }\left(a|s\right)\right]. \end{array}$$

The λ in Equation (10) should decay to zero, promoting generalization to new environments. If the new environment is more different from where the demonstration is provided, smaller initial λ or larger decay factor should be chosen. To refine the result policy and avoid compounding errors due to the visited state distribution mismatch between the compound policy and the child policy, we run the result child policy for one episode to collect new samples to aggregate training set. With that training set, we optimize Equation (10) with the compound policy as supervisor. Then, with new child policy, the data collection and optimization processes can be executed again. This procedure can be iterated for several times and generates a final child policy that performs well under its own induced state distribution. And it is similar to the imitation learning with Dataset Aggregation (DAgger) algorithm (Ross et al., 2011). The KL-divergence in Equation (10) promotes high entropy in result policy, and thus encourages the exploration too. For Gaussian distribution, the surrogate loss (Equation 10) is easily optimized with stochastic gradient descent. The crossover operator in EPG is very sample-efficient. In experiments, we only iterated the Dataset Aggregation procedure for two or three times. The whole procedure of crossover operator is shown in Algorithm 2.

## 5. Experiments

In the first part of this experiment section, we present the improved exploration capability of our method, comparing with the state-of-the-art reinforcement learning methods, where no demonstrations are provided. In the second part, we demonstrate the generalization capability of our method to different situations in robot learning from demonstration, where robot accommodates to new environments with active exploration. And we present that our method has better performance in generalization, in comparing with other related methods which combining imitation learning and reinforcement learning.

### 5.1. Exploration: Robot Control in State Space

Reinforcement Leaning methods are well-known as its capability of exploration in unknown environments without any instruction or demonstration from “expert.” To present the improved exploration of our EPG, we set λ = 0 in Equation (10) and compare our EPG-PPO with the state-of-the-art reinforcement learning methods, ES and DPPO, in continuous control problems of OpenAI Gym (Brockman et al., 2016) without demonstrations. For comparison with related works and future research, we choose the average cumulative rewards, provided by the simulator in OpenAI Gym, as the evaluation criterion.

In our experiments we implement our EPG with PPO (Schulman et al., 2017) as policy gradient method, called EPG-PPO. DPPO is the abbreviation of “distributed proximal policy optimization” method (Heess et al., 2017) which is popular with its sample efficiency. In DPPO the distributed agents explore in environments with different random seeds and calculate the policy gradients which are averaged to update the policy. Our implementation of DPPO has the same full connected neural network structure as that of our EPG-PPO, composed with two hidden layers and “tanh” activation function. The dimension of each layer is ten times of the dimension of each robot's action space. And other major hyperparameters are similar, e.g., 2–5 nodes in parallel and learning rates within [0.0005, 0.001]. ES is the abbreviation of “evolutionary strategy” (Salimans et al., 2017) which is good at random exploration. In our implementation the policy is perturbed to form 5-8 individuals in population to generate experience and is updated according to the evaluation of those experience.

**Figure 2:**
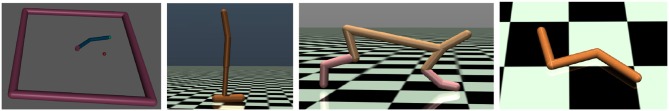
Environments for the Robot Control in State Space: “Reacher,” “Hopper,” “HalfCheetah,” and “Swimmer”.

**Figure 3:**
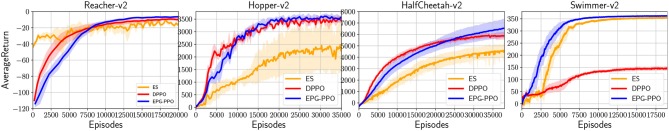
Depict the average cumulative rewards of the policy after the crossover in EPG-PPO, the distributed policies in DPPO and the updated policy in ES, plotted over the episodes required during training. The solid lines represent the average performance in six repeated experiments with different random seeds and the shades exhibit the standard deviation of the performance in those repeated experiments.

In some standard OpenAI Gym environments, such as “Reacher,” “Hopper,” “HalfCheetah,” and “Swimmer,” robots will definitely receive a task-related reward signal at each state with its value dependent on the state and action. Figure 2 illustrate the scenes of robot control tasks in our experiments. In the first picture, the goal of “Reacher” is to control a two-arm robot with torque so as the end-effect approach the target point as near as possible, in 50 time steps. In the other three pictures, “Hopper,” “HalfCheetah,” and “Swimmer” are multi-joint robots which get more rewards by running or swimming forward away. Figure 3 depicts the comparative performance in environments with dense rewards. Especially in “Swimmer,” our EPG avoids the local minima which traps the PPO.

**Figure 4:**
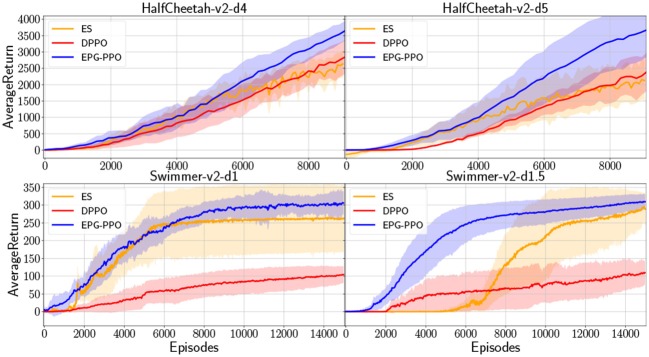
In environments with sparse rewards, “HalfCheetah-v2-d4” and “HalfCheetah-v2-d5” provide reward signal until HalfCheetah's position in *x* axis surpass 4 and 5 units, respectively. “Swimmer-v2-d1” and “Swimmer-v2-d1.5” are modified with rewards delayed for 1 and 1.5 units in *x*, respectively.

In many real-world problems, robots may only receive rewards after the task accomplishment, where exploration play a more important role in robot learning. To construct environment with sparse reward, we modify the original gym environments, “HalfCheetah-v2” and “Swimmer-v2,” to be with no reward at the beginning until the robot walk forward to surpass a threshold. In such situation robots must explore more to acquire reward signal and learn to accomplish the goal which, here in our experiments, is running forward as far as possible. The comparative results are illustrated in Figure 4, which depicts the performance of EPG-PPO, DPPO and ES, with the solid lines and shades representing the mean and standard deviation in six repeated experiments. In those environments with sparse rewards, the agents are initialized with random orientations and acquire performance with high variance in repeated experiments. Although three algorithms all have worse performance than that in Figure 3 due to the sparse reward, our EPG has more advantage with regard to the sample efficiency.

### 5.2. Generalization: Learning Vision Based Manipulation From Demonstration

**Figure 5:**
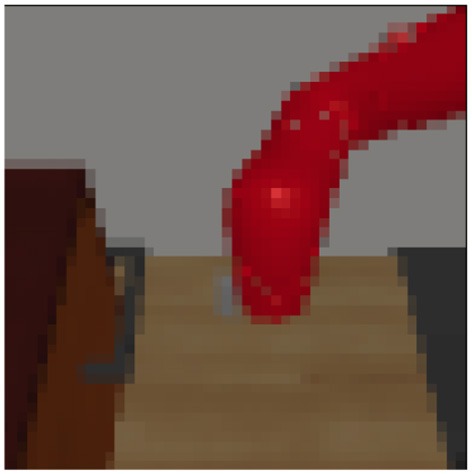
Robot view.

To present the generalization of our EPG, in the complex manipulation task, we use simulation environment “Image48SawyerDoorPullHookEnv-v0” provided in “multiworld”^1^. In this task, the demonstrations, including images and corresponding displacements of the end effector, are collected in original environment, while robot is learning and tested in different environments with distinct configurations of the door. The goal of this task is to open the door in 100 time steps. We train a neural network policy, which is composed with two convolutional layers and two full connected layers with 100 units for each. And we use “tanh” as the activation functions in hidden layers. The policy takes as input the images from the fixed camera and outputs the next displacement of the end-effector. The image viewed by the robot is with 48 × 48 pixels, shown in Figure 5. The robot receives a binary reward when the angle of door is above a threshold.

**Figure 6:**
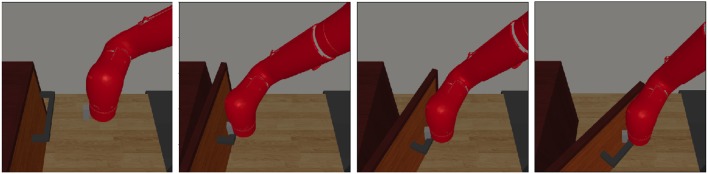
Neural network policy trained with our EPG accomplish the task.

**Figure 7:**
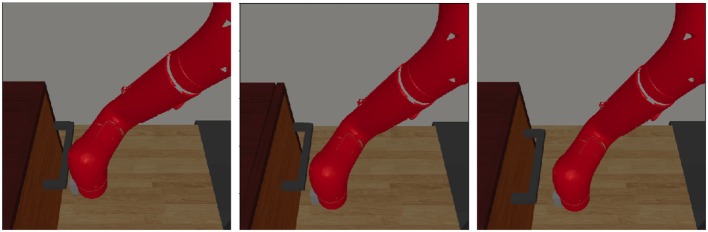
Neural network policy trained with behavior clone failed.

In the initial environment, we hand-engineer the robot to open the door and collect only one trajectory including images and the corresponding actions. We augment the demonstration dataset by adding minor noises to the actions with the images copied. Then, we change the configuration of the door, such as the position, initial angle and its handle's position. In the new situation and with our EPG-PPO, robot interacts with the environment about thousands of time steps and learn from demonstration at the same time, robot can open the door with high success rate, as illustrated in Figure 6. While the robot learned with behavior clone without exploration failed in adapting to the new situation as depicted in Figure 7.

Even though we do not find any previous works aiming at generalizing skill learning from demonstration to different scenarios where the demonstrations are provided, the reinforcement learning after imitation learning is a good thought to accomplish such goal. To present the better generalization capability of our method, we implement “BC + DPPO” as baseline, where the robot is first trained with behavior clone to learn from demonstration, then trained with DPPO to generalize by exploration in new environments.

**Figure 8:**
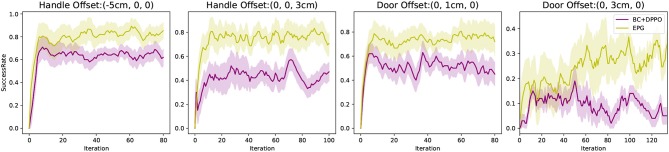
Success rate comparisons with other method in variant scenarios.

Figure 8 shows our experiments, where we change the positions of the door and its handle with an offset relative to their origins. In those experiments, we compare our EPG with “BC + DPPO.” It is obvious that with the same few demonstrations, our EPG can learn from demonstration and generalize better. Even though the robot learns from the binary reward signal it receives, we evaluate our method by calculating the success rate of the task accomplishment, i.e., door is open, in 100 independent tests during the process of training. Figure 8 shows the mean values and standard deviations of the success rates in five repeated experiments.

## 6. Conclusion and Discussion

In this paper we introduced a new learning from demonstration method, Evolutionary Policy Gradient (EPG), with demonstration guiding the evolution of policy. Just providing the demonstrations from one specific situation, our EPG can generalize the robot learning from the demonstrations to accomplish the same tasks in different environments.

With parameter perturbation and evolutionary framework, our EPG explore in the new environment to accomplish the task. With demonstration guiding and policy gradient optimization, robot can acquire the skill to accomplish the task with fewer interaction with the new environment. In the framework of EAs, EPG is scalable in parallel to accelerate the training process. Moreover, our EPG is a general framework and can be implemented with all kinds of policy gradient methods. The whole optimization procedure of EPG is based on the stochastic policy gradient theorem and behavior clone, with a little approximation to fit the framework of EAs.

Our aim is to improve the generalization of robot learning skill from demonstration. As the main contribution of our work, we present that the active exploration of robot can accomplish the goal of generalization. Next, we discuss the rationality behind our study from the perspectives of exploration, exploitation and generalization.

### 6.1. About Exploration

As a well-known Evolutionary Algorithm, evolutionary strategy (ES) only relies on exploring with parameter perturbation. When the reinforcement learning problem is with dense reward, our results shown in Figure 3 demonstrated that ES has few advantage over DPPO which exploit the powerful policy gradient methods. On the other hand, ES outperforms DPPO in environment with sparse reward. Our results shown in Figure 4 illustrate that parameter perturbation has more advantage in exploration. And, our EPG-PPO explores with parameter perturbation and retains the data efficiency of policy gradient. Thus, our method combines the merits of both ES and DPPO.

### 6.2. About Exploitation and Exploration Trade-Off

Our policy parameter perturbation with Gaussian noise is also a sample from Gaussian distribution with the optimized mean and annealing variance. The policy parameter sampling after every optimization step can be seen as a posterior sampling. From this point of view, our EPG is an approximation to Thompson Sampling (Thompson, 1933) in the policy parameters. Thompson Sampling, originated from bandits problems, provides an elegant approach that tackles the exploration-exploitation dilemma. Previous works, inspired by Thompson Sampling, focus on problem with discrete action space by randomly selecting an action according to the probability it is optimal. Our method are aiming at problems with continuous action space. Improving our method toward the Exploration and Exploitation trade-off is a promising direction for future research. For example, we can take into the parameter variance optimization into consideration, making the parameter perturbation procedure bears more similarity to Thompson Sampling.

### 6.3. About Generalization

In the unknown environment without demonstration, robot trained with many state-of-the-art reinforcement learning methods, including DPPO, can learn to accomplish many complex tasks by interacting with the environment millions of time steps. In our experiments, we find that DPPO can not make robot explore to grasp the skill of opening the door in thousands of interaction time steps, due to the sparse reward. Exploration from scratch is sample inefficient and can not be seen as generalization, though with adaption to new environments. Our method learning from demonstration and adapting to new environments with few interactions are actually generalizing the learning skill from previous situation to new situation. Our deep combination of learning from demonstration and reinforcement learning presents a promising direction to improve the generalization of learning from demonstration and is worth further research. On the perspective of sample complexity, it is the generalization from demonstration that makes the reinforcement learning process more sample efficient. Thus, our method can also provide a new method to improve the sample efficiency of Reinforcement Learning methods.

## Data Availability Statement

Publicly available datasets were analyzed in this study. This data can be found here: https://github.com/vitchyr/multiworld, https://github.com/openai/gym.

## Author Contributions

JC, WL, YL, and JY conceived the project. YL and JY acquired funding. JC coordinated the subprojects and wrote the manuscript. JC and WL conducted the experiments, analyzed the data, and prepared figures.

## Conflict of Interest

The authors declare that the research was conducted in the absence of any commercial or financial relationships that could be construed as a potential conflict of interest.

## Footnotes

^1^https://github.com/vitchyr/multiworld
